# Feruloyl esterase (FAE-1) sourced from a termite hindgut and GH10 xylanases synergy improves degradation of arabinoxylan

**DOI:** 10.1186/s13568-021-01180-1

**Published:** 2021-01-19

**Authors:** Mpho S. Mafa, Samkelo Malgas, Brett I. Pletschke

**Affiliations:** 1 Enzyme Science Programme (ESP), Department of Biochemistry and Microbiology, Rhodes University, Grahamstown, 6140 South Africa; 2 Department of Plant Sciences, University of the Free State, P.O. Box 339, Bloemfontein, 9300 South Africa; 3 Department of Biochemistry, Genetics and Microbiology, University of Pretoria, Hatfield, 0028 South Africa

**Keywords:** Arabinoxylan, Hemicellulose, Feruloyl esterase, Glycoside hydrolase, Enzyme synergy, Xylanase

## Abstract

Cereal feedstocks have high arabinoxylan content as their main hemicellulose, which is linked to lignin by hydroxycinnamic acids such as ferulic acid. The ferulic acid is linked to arabinoxylan by ester bonds, and generally, the high substitution of ferulic acid leads to a loss of activity of xylanases targeting the arabinoxylan. In the current study, a feruloyl esterase (FAE-1) from a termite hindgut bacteria was functionally characterised and used in synergy with xylanases during xylan hydrolysis. The FAE-1 displayed temperature and pH optima of 60 ℃ and 7.0, respectively. FAE-1 did not release reducing sugars from beechwood xylan (BWX), wheat arabinoxylan (WAX) and oat spelt xylan (OX), however, displayed high activity of  164.74 U/mg protein on *p*-nitrophenyl-acetate (pNPA). In contrast, the GH10 xylanases; Xyn10 and XT6, and a GH11 xylanase, Xyn2A, showed more than two-fold increased activity on xylan substrates with low sidechain substitutions; BWX and OX, compared to the highly branched substrate, WAX. Interestingly, the FAE-1 and GH10 xylanases (Xyn10D and XT6) displayed a degree of synergy (DS) that was higher than 1 in all enzyme loading combinations during WAX hydrolysis. The 75%XT6:25%FAE-1 synergistic enzyme combination increased the release of reducing sugars by 1.34-fold from WAX compared to the control, while 25%Xyn10D:75%FAE-1 synergistic combination released about 2.1-fold of reducing sugars from WAX compared to controls. These findings suggest that FAE-1 can be used in concert with xylanases, particularly those from GH10, to efficiently degrade arabinoxylans contained in cereal feedstocks for various industrial settings such as in animal feeds and baking.

## Introduction

Ferulic acid cross-linking of xylan to lignin restricts Carbohydrate-Active Enzymes (CAZymes) such as xylanases and cellulases from degrading structural polysaccharides efficiently and, as a result, limits the utilisation of agricultural crop residues as (1) feeds for animals by shielding the non-starch polysaccharide from the xylanolytic hydrolytic activity; (2) it limits feedstocks uses for biofuel by hindering the biomass access to holo-cellulolytic enzymes (xylanases and cellulases); and (3) affects the production of value-added chemical products such as xylo- and cello-oligosaccharides (Grabber et al. 1998). Agricultural cereal crops such as maize, wheat, oat and barley are monocotyledons (monocots) that belong to the Poaceae family (Chung and Hoang 2019, Schendel et al. 2015 and Schendel et al. 2016). Xylan is a major hemicellulosic component of monocots/agricultural crops, which is generally linked to lignin by hydroxycinnamic acids such as ferulic acids (FA) and of *p*-coumaric acids (CA) (Mnich et al. 2020).

In general, xylan consists of a linear backbone chain of β-(1,4)-linked xylose units substituted with acetyl, D-glucuronic acid (GlcA) and/or 4-*O*-methyl-D-glucuronic acid (Me-GlcA), and L-arabinose residues. Agricultural feedstock sources such as maize, wheat, oat, rice and barley consist of arabinoxylans (AX) and glucuronoarabinoxylans (GAX) that are also linked to ferulic acid by ester bonds via the branching arabinose residues (Boz 2015; Mnich et al. 2020 and Malgas et al. 2019). According to Mnich et al. (2020), ferulic acid and its oligomeric forms are also vital components of the plant cell because they link the xylan to lignin. The β-1,4-linked xylopyranosyl linear polymer is generally substituted at hydroxyl groups attached to the second or third carbon (C2-O- and/or C3-O-). In addition, AX or GAX are linked to ferulic acid, ferulic acid dimers, or coumaric acid via ester bonds which are formed at the fifth carbon hydroxy group (C(*O*)5-hydroxy group) of α-L-arabinosyl residues (Grabber et al. 2000; Faulds et al. 2003 and Mnich et al. 2020). The branched heterologous nature of the ferulated-xylans impedes the hydrolytic potential of xylanases. Hence, we propose that it is important to establish how the synergy between different xylanases and feruloyl esterases (FAE) may improve the degradation of these xylans contained in agricultural feedstocks.

There has been a great interest in research towards the extraction or production of hydroxycinnamic acids (particularly FA and CA) from agricultural feedstocks using chemical or enzymatic means (Mkabayi et al. 2020 and Yu et al 2002). For instance, Yu et al. (2002) used alkali pretreatment to extract about 3.83 µg mg^−1^ dry matter of ferulic acid from oat hull. The cell walls extracted from maize cell suspensions contained about 18 µg mg^−1^ of dry matter of ferulic acid (Grabber et al. 1998). In addition, Mkabayi et al. (2020) used two feruloyl esterases (FAE) referred to as FAE5 and FAE6 in combination with a GH11 xylanase to produce about 2–3 µg/ml ferulic acid from wheat-flour arabinoxylan and corncob. The extraction of the ferulic acid from cereal crops by alkali treatments or enzyme technologies is well-established, however, there are a few studies that sought to understand how FAEs synergistically interact with xylanases from glycoside hydrolase (GH) family 10 and 11. The current study attempted to elucidate the FAE synergy with the well-characterised GH10 and GH11 xylanase enzymes.

Due to their different xylan cleaving sites, GH10 and GH11 xylanases have been used synergistically for improved hydrolysis of the xylan biomass (Beaugrand et al. 2004). Several studies performed synergy   assays with xylanases and cellulases (van Dyk and Pletschke 2012), xylanases and debranching enzymes, i.e. arabinofuranosidases (Xin et al. 2019), xylanases from GH8, GH10 and GH11 for improved xylan hydrolysis (Malgas and Pletschke 2018). However, very few studies have investigated the interaction of the xylanases and the FAEs. Mkabayi et al. (2020) investigated the synergistic use of termite derived FAEs with a GH11 xylanase for production of hydroxycinnamic acid, while Rahmani et al. (2018) studied xylanase and FAE from actinomycetes cultures for improved hydrolysis of the sugarcane bagasse for the release of fermentable sugars.

We argue that for the past decade the focus of research regarding lignocellulolytic biomass hydrolysis was on the synergy between cellulases and xylanases or xylanases and debranching enzymes, but little focus was given to the role of FAE enzymes on biomass hydrolysis. Therefore, the current study attempts to establish synergistic interactions between different GH10 and GH11 xylanases with a termite-metagenome derived FAE-1 during the degradation of wheat, oat and beechwood xylan.

## Methods and material

### Substrates and enzymes

Two GH10 xylanases, XT6 from *Geobacillus stearothermophilus* and Xyn10D from *Cellvibrio japonicus*, were purchased from the Megazyme (Bray, Ireland), while the GH11 referred to as Xyn2A from *Trichoderma viride* was purchased from Sigma Aldrich (St. Louis, USA). Insoluble wheat arabinoxylan (WAX) and beechwood xylan (BWX) substrates were purchased from Megazyme (Bray, Ireland) and *p*-nitrophenyl acetate (*p*-NP-acetate) and oat spelt xylan (OX) were purchased from the Sigma Aldrich (St. Louis, USA). The purified feruloyl esterase from family 1 (FAE-1) with the following GenBank accession number KC493563 derived from the termite hindgut (*Trinervitermes trinervoides*) was provided by Dr. K. Rashamuse, Council for Scientific and Industrial Research (CSIR) Biosciences (Pretoria, South Africa). FAE-1 was expressed and purified according to the method described by Rashamuse et al. (2014).

### Protein determination

Protein concentration of the enzymes was measured using the Bradford’s method (Bradford 1976). Bovine serum albumin was used as a suitable protein standard.

### Enzyme activity determination

The substrate specificities of the xylanases (XT6, Xyn10D, and Xyn2A) and FAE-1 were determined under standard assay conditions (pH 7.0, 37 °C temperature and continuous rotation at 25 rpm). For the xylanase activity assays, 400 µl of 1% (w/v) WAX, BWX and OX substrates were dissolved or suspended in 50 mM sodium phosphate buffer and 10 µg of enzyme was used. The reaction time for the xylanase activity was 30 min and then the reaction was terminated by heat at 100 ℃ for 5 min. The total reducing sugars released by the enzymes from xylan substrates were determined spectrophotometrically at 540 nm using the modified dinitrosalicylic acid (DNS) method as described by Miller (1959). Xylose was used as a suitable standard to determine the total reducing sugars. For FAE activity determination, 2.5 mM *p*NP-acetate was dissolved in 50 mM sodium phosphate buffer and the 15 min reaction was initiated by the addition of 10 µg enzyme load. The reaction was quenched by the addition of 2 M NaCO_2_ and the release of *p*-nitrophenol read spectrophotometrically at 410 nm. The pH optimum of FAE-1 was measured by varying the pH of the reaction from 3 to 9 using a universal buffer (50 mM Tris, 50 mM boric acid, 33 mM citric acid, and 50 mM Na_2_PO_4_ adjusted with either HCl or NaOH to the required pH) (Britton and Robinson 1931), while the temperature optimum was determined by incubating the reactions at temperatures between 30 and 80 ℃. All experiments were performed in quadruplicate.

### Synergy studies

Binary enzyme mixtures were formulated using the individual xylanases (Xyn10D or XT6 and/or Xyn2A) and FAE-1 to evaluate their synergistic associations during the hydrolysis of 1% (w/v) BWX, WAX and OX. The enzyme combination ratios of 100:0%, 75:25%, 50:50%, 25:75% and 0:100% on protein mass basis at 10 mg/g of xylan were used to investigate the synergism between enzymes. The reaction was initiated by mixing 1% (w/v) xylan (BWX, WAX and OX) with the binary enzyme mixtures in 50 mM sodium phosphate buffer (pH 6.0) at 37 °C and run for 12 h. The reducing sugar release was then analysed according to DNS method described in the “Enzyme activity determination” section. All experiments were performed in quadruplicate.

### Determination of the degree of synergy (DS)

The degree of synergy (DS) was defined as the total reducing sugars produced by synergistic actions of the two or more enzymes divided by the theoretical sum of total reducing sugars produced by the individual enzymes (van Dyk and Pletschke 2012; Mafa et al. 2020). To determine the DS between the individual xylanases and FAE-1 the reducing sugars released by binary varying combinations of 0%, 25%, 50%, 75% and 100% were detected as described in “Synergy studies” section. The data was used to calculate the DS according to the following equation:

1 $$DS = \frac{TRS\;released\;by\;a\;combination\;of\;A:B}{{\left( {TRS\;released\;by\;A} \right) + \left( {TRS\;released\;by\;B} \right)}}.$$

DS represents the degree of synergy; TRS represents the total reducing sugars produced by lone enzyme A or B and/or a combination of A:B, where A and B represent combinations of xylanase and FAE-1.

### Data analysis

One-way analysis of variance (ANOVA) was used to elucidate significant differences in the reducing sugar released by different binary enzyme mixtures referred to in “Synergy studies” and “Determination of the degree of synergy (DS)” sections. All pairwise comparisons were conducted using the Data analysis of Microsoft® Excel 2013.

## Results

### Specific activity and characterization

#### FAE-1 specific activity and biochemical properties

The focus of the current study was to (1) demonstrate that the FAE-1 derived from a termite hindgut bacteria is active; (2) biochemically characterise the FAE-1; and (3) formulate binary FAE-1 to xylanase synergistic enzyme mixtures to improve the release of reducing sugars during xylan hydrolysis.

**Fig. 1:**
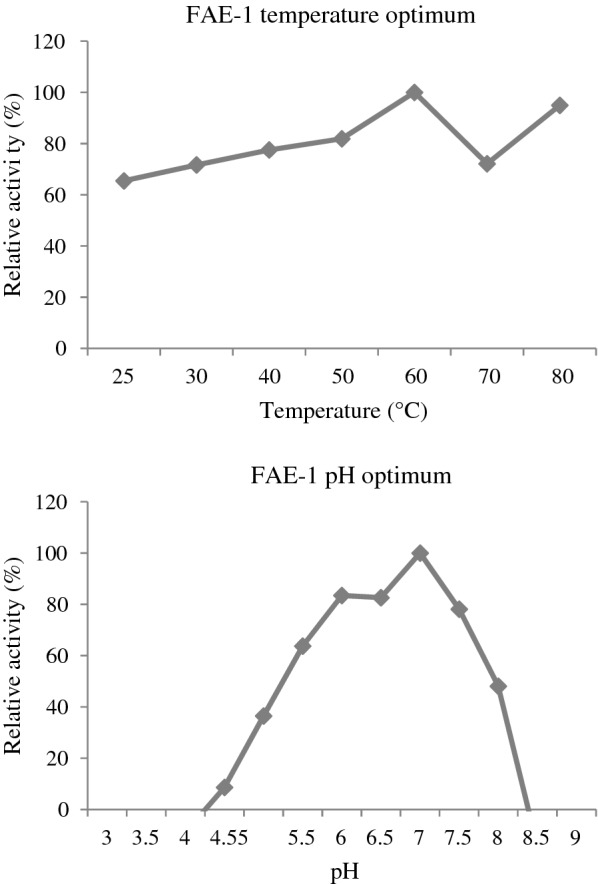
The temperature and pH optimum of FAE-1. The release of *p*-nitrophenol was used to determine the relative activity during the hydrolysis of *p*NP-acetate. Values are represented as mean values ± SD (n = 3)

FAE-1 showed no activity on all the xylan substrates (BWX, WAX and OX), however, it displayed a high specific activity of about 164.74 U/mg protein on the *p*-NP-acetate (Table 1). Figure 1 shows that FAE-1 optimum pH was at pH 7.0, however, it also showed more than 80% of relative activity at pH 6.0 to 6.5. The temperature optimum of FAE-1 was recorded at 60 ℃. Between 50 and 70 ℃, the enzyme showered more than 80% relative activity.

**Table 1:** Enzyme specific activity (U/mg protein) for FAE and xylanolytic enzymes

Substrates	Individual enzymes analysed			
	FAE-1	Xyn10D	XT6	Xyn2A
Beechwood xylan	0	125.42 ± 0.15	128.84 ± 0.19	170.30 ± 0.07
Wheat arabinoxylan	0	56.67 ± 0.06	60.24 ± 0.06	73.44 ± 0.04
Oat spelt xylan	0	156.19 ± 0.19	121.46 ± 0.12	212.11 ± 0.12
*p*NP-acetate	164.74 ± 0.016	ND	ND	ND

FAE-1 represent the termite (*T. trinervoides*) hindgut derived feruloyl estarase; Xyn10D represent GH10 xylanase from *C. japonicus*; XT6 represent GH10 xylanase from *G. stearothermophilus*, and Xyn2A represent GH11 xylanase from *T. viride*. U = µmol/min/mg protein for FAE-1 or µmol/h/mg proteins for xylanolytic enzymes; ND = not tested

#### Xylanase specific activity

**Fig. 2:**
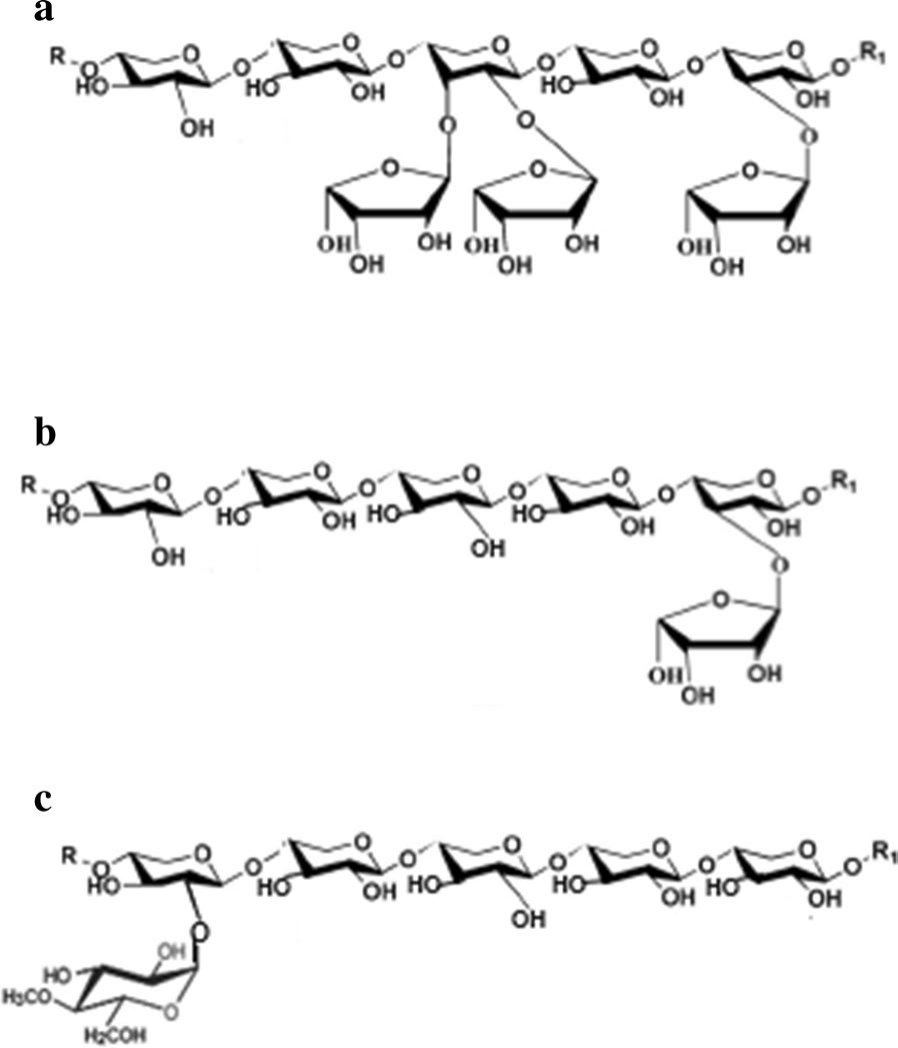
The chemical structures of xylan from different sources. Xylan consists of the backbone chain of xylose units joined by β-1,4-glycosidic bond, and substituted by α-arabinofuranosyl residues at second or third carbon (C2-O- and/or C3-O-). The xylan backbone chain extension from the non-reducing end is represented by R and extension from reducing ends are represented by R1. The wheat arabinoxylan used in the current study was highly substituted as indicated in **a** and oat arabinoxylan had lesser substitution as indicated in **b**. Glucuronoxylan consists backbone of xylose units β-1,4-d-glycosidic bond that is substituted with 4-*O*-methyl-α-d-glucopyranosyl uronate residues. Beechwood xylan used in the current study consisted mostly of glucuronoxylan as indicated in **c**

The xylanases (Xyn10D, XT6 and Xyn2A) were tested on the xylan substrates; BWX, WAX and OX. Xyn10D and Xyn2A showed the highest specific activity on OX reaching about 156.19 and 212.11 U/mg protein, respectively. XT6 showed the highest specific activity of 128.84 U/mg protein on BWX (Table 1). All three xylanases displayed the lowest enzyme activity on WAX. We argue that the chemical structure and the sidechain substitution frequencies of BWX, WAX and OX were the major contributing factors to the varied xylanase activity on these substrates. The Megazyme composition sheet of BWX (Lot 171002; CAS No. 9014-63-5) and WAX (Lot 120801c; CAS No. 9040-27-1) demonstrate that BWX is composed of about 80.8% xylose and 11.4% glucuronic acid, while WAX consist of about 36% arabinose, 51% xylose, 6.5% glucose, 4.4% mannose and 1.6% galactose. On the other hand, Chung and Hoang (2019) revealed that OX is composed of 73.4% xylose, 6.6% arabinose and 0.4% galactose. From this data, it was evident that WAX was the most substituted xylan substrate, consisting of about 30% more arabinose residues compared to OX. For every five xylose units of the backbone xylan, there were three substituted arabinose units in WAX (Fig. 2a). Interestingly, BWX and OX were less substituted and displayed a sugar composition ration of 8.8:1.14:0.78 and 7.34:0.66:0.04 (i.e. for every 7 xylose units there was about 1 arabinose unit in OX or for every 9 xylose units there was 1 4-*O*-methyl-α-d-glucopyranosyl uronate residues in BWX; Fig. 2b, c). Surprisingly both GH10 (Xyn10D and XT6) and GH11 (Xyn2A) were highly active on the less substituted substrates (Table 1). The biochemical properties of the xylanases were previously verified by our research group (Table 2). The biochemical properties of the xylanases and FAE-1 revealed that these enzymes could be use in synergy because they displayed more than 80% relative activity at 40 ℃ and pH 6.0. These reaction parameters were then chosen for the subsequent synergy studies as standard reaction conditions.

**Table 2:** Biochemical properties of the commercial xylanolytic enzymes

Enzymes	pH optimum	Temperature optimum (℃)	GH families	References
XT6	6.5	70	10	Megazyme (Lot 101003e); Malgas and Pletschke (2018)
Xyn10D	5	60	10	Megazyme (Lot 131101b)
Xyn2A	6	50	11	Malgas and Pletschke (2018)

All xylanolytic enzymes displayed pH stability over a range of pH 5–8

### Binary enzyme synergy

It was established in the past decade that some CAZymes (http://www.cazy.org/Glycoside-Hydrolases) can be used in value-added chemical production, baking and feed industries. A few CAZymes (xylanases) that degrade the non-starch polysaccharides (NSPs) were generally used in the baking and feed industry. We propose that understanding the synergy between CAZymes during the hydrolysis of NSPs could lead to improved degradation of feedstocks containing NSPs. Hence, in the current study, binary synergy between (1) Xyn10D and FAE-1; (2) XT6 and FAE-1; and (3) Xyn2A and FAE-1 was investigated for improved hydrolysis of xylans.

#### GH10 (XT6) and FAE-1 synergy

**Fig. 3:**
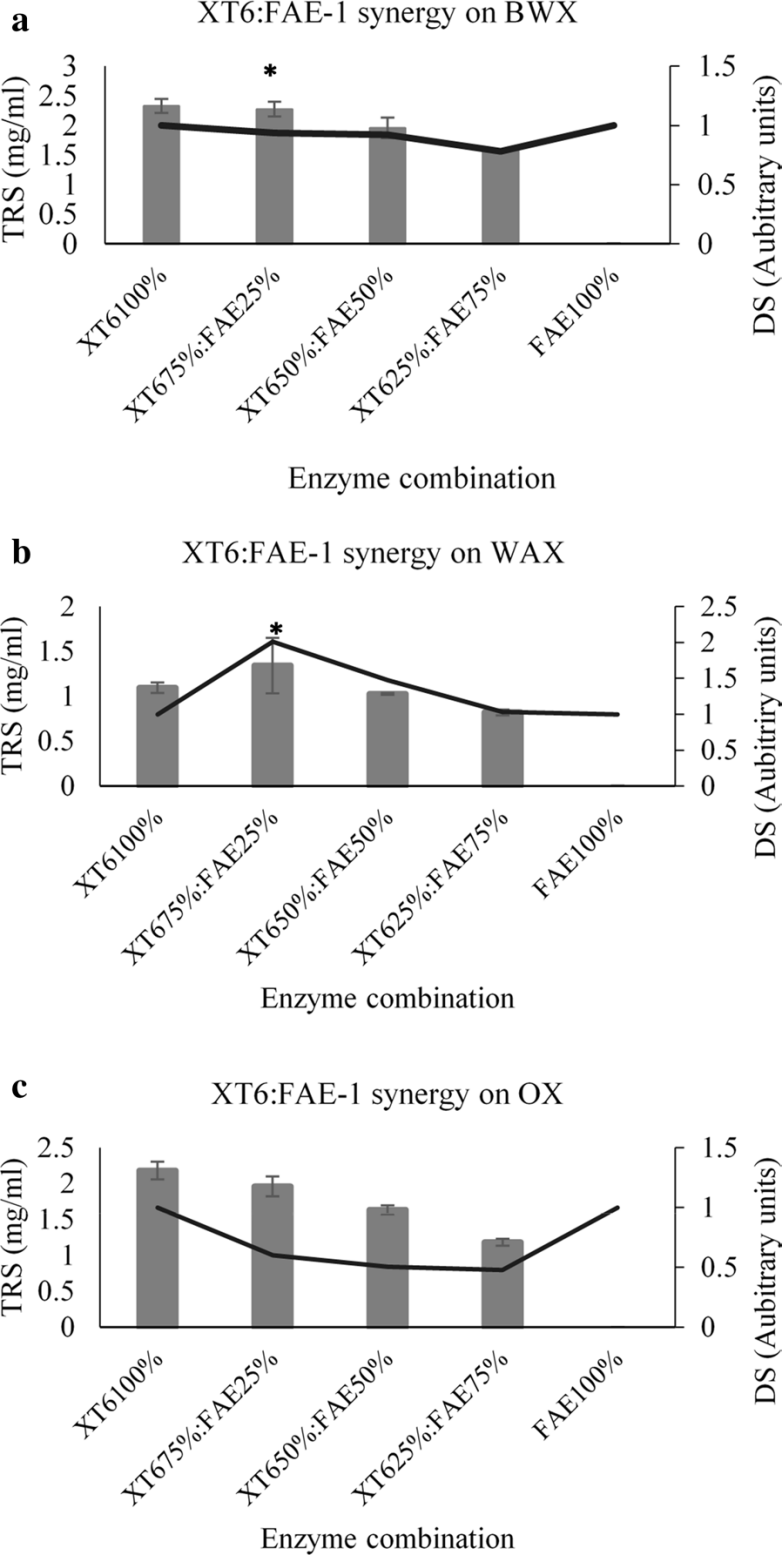
Synergistic hydrolytic activities of the GH10 xylanase (XT6) and feruloyl esterase (FAE-1) on beechwood xylan (**a**), insoluble wheat arabinoxylan (**b**) and oat spelt xylan (**c**). The TRS represent the amount of released total reducing sugars from xylan and DS represents the degree of synergy formed between two enzymes. ANOVA analysis indicated the improvement of reducing sugar release by the enzyme combinations compared to lone enzyme protein loading, keys: *(p value < 0.05) and #(p value < 0.01). Values are represented as mean values ± SD (n = 3)

The BWX xylan is substituted with 4-*O*-methyl-α-d-glucopyranosyl uronate residues and lower amounts of acetyl groups. Therefore, the absence of the ferulic acid or *p*-coumaric acid in BWX suggests that synergy between the xylanases and FAE-1 could only occur if FAE-1 also possesses acetyl xylan esterase activity. We expected to see synergy between xylanases and FAE-1 during the hydrolysis of the WAX and OX since these substrates are derived from cereals which generally contain cinnamic acids linked to xylans. The degree of synergy (DS) is one of the most powerful tools used in establishing synergism between enzymes (Van Dyk and Pletschke 2012, and Mafa et al. 2020) and it was employed in the current study to investigate synergism between xylanases and FAE-1. Figure 3a shows that a DS of about 1 was recorded between XT6 and FAE-1 at an enzyme load of 75%:25% during BWX hydrolysis. A DS greater than 1 reveals that there was synergism between these enzymes. At other enzyme ratio combinations, the DS was less than 1, which suggests that there was no synergism between XT6 and FAE-1 during the hydrolysis of BWX by these combinations. The decrease in released reducing sugars during BWX hydrolysis when the combination was at 50% XT6 to 50% FAE-1 (1.96 mg/ml) and 75% XT6 to 25% FAE-1 (1.57 mg/ml) was also indicative of the fact that there was no synergy between the two enzymes.

Interestingly, XT6 and FAE-1 displayed synergism in all the enzyme combinations during the degradation of WAX as the DS values were above 1 (Fig. 3b). The degree of synergy reached the highest value of 2.01 and released about 1.23-fold higher reducing sugar (1.4 mg/ml) at an enzyme loading of 75% XT6 to 25% FAE-1 compared to 100% XT6 that only released 1 mg/ml of reducing sugars. At the enzyme loading of 50% XT6 to 50% FAE-1, the enzymes released about 1. 02 mg/ml reducing sugars from the WAX substrate and displayed a DS of about 1.5. Figure 3c shows that there was no synergism between the XT6 and FAE-1 during the hydrolysis of OX. The decrease in XT6 enzyme load from 100 to 25% resulted in about 50% drop in the released reducing sugars from 2.18 mg/ml (100% XT6) to 1.17 mg/ml (25%XT6:75%FAE-1).

#### GH10 (Xyn10D) and FAE-1 synergy

**Fig. 4:**
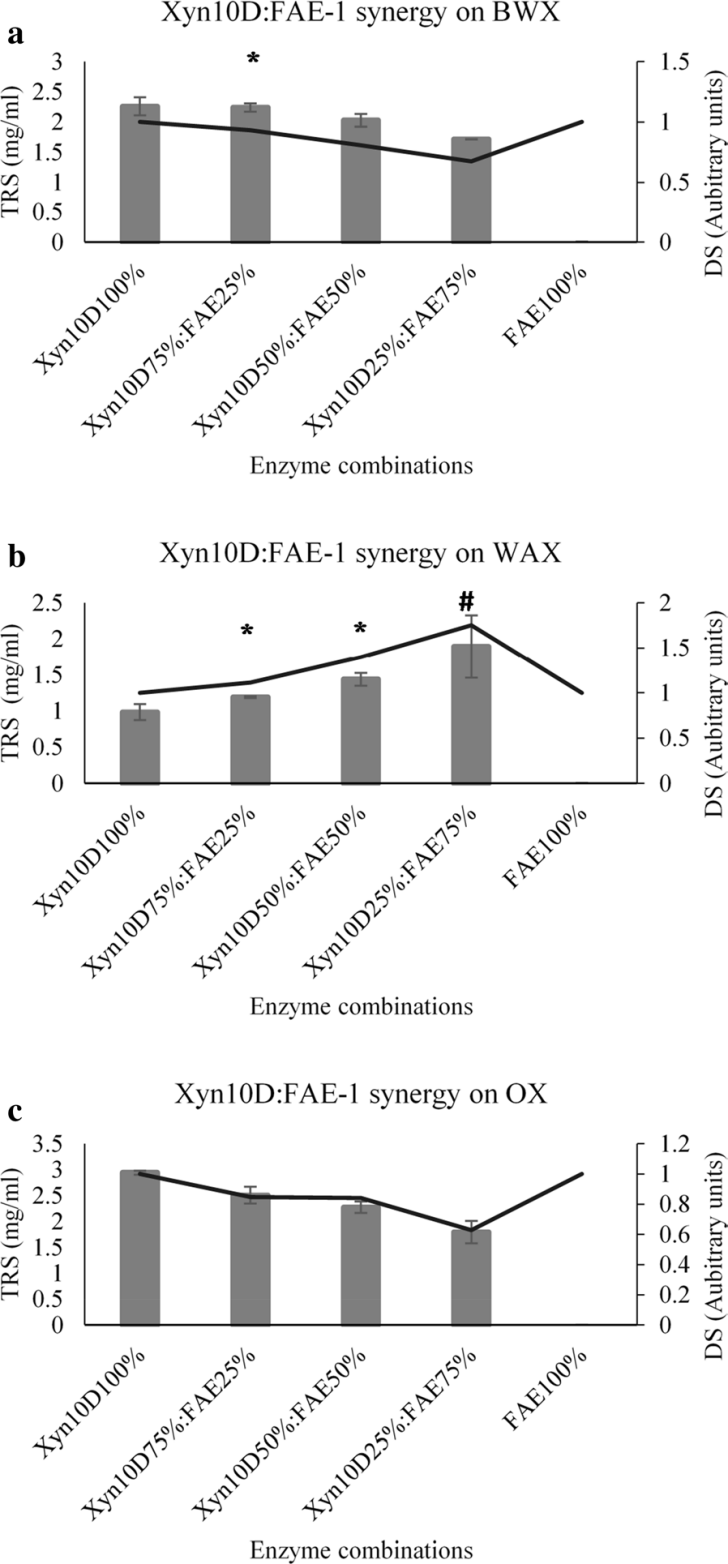
Synergistic hydrolytic activities of the GH10 xylanase (Xyn10D) and feruloyl esterase (FAE-1) on beechwood xylan (**a**), insoluble wheat arabinoxylan (**b**) and oat spelt xylan (**c**). The TRS represent the amount of released total reducing sugars from xylan and DS represents the degree of synergy formed between two enzymes. ANOVA analysis indicated the improvement of reducing sugar release by the enzyme combinations compared to lone enzyme protein loading, keys: *(p value < 0.05) and #(p value < 0.01). Values are represented as mean values ± SD (n = 3)

The synergy between Xyn10D and FAE-1 was investigated during the hydrolysis of xylans. Xyn10D and FAE-1 displayed a DS of about 0.95 during the hydrolysis BWX, which is arguably close to 1 and suggest that a combination of 75% Xyn10D and 25% FAE-1 resulted in no synergism. The released reducing sugars demonstrated that 100% Xyn10D release about 2.5 mg/ml reducing sugars, which was not significantly different from 2.4 mg/ml reducing sugars released by 75%Xyn10D:25%FAE-1 (Fig. 4a). However, at the combination 50%Xyn10D:50%FAE-1 and 25%Xyn10D:75%FAE-1 displayed DS lower than 1, which suggested that there was no synergy between the enzymes. These findings were supported by the decrease in released reducing sugar 2.0 mg/ml (50%Xyn10D:50%FAE-1) and 1.7 mg/ml (25%Xyn10D:75%FAE-1). Figure 4b shows a much more interesting phenomenon of a linear increase in the DS values of Xyn10D and FAE-1, and gradual increase in released reducing sugars by the combined hydrolytic activities of the Xyn10D and FAE-1. The highest DS value of about 1.75 was recorded at a combination of 25%Xyn10D:75%FAE-1 and the reducing sugars were about twofold higher than the control at this combination. Given that the 100% FAE-1 did not release the reducing sugars we propose that the FAE-1 could degrade the ester bonds linking ferulic acid or *p*-coumaric acid to arabinoxylan, hence, FAE-1 synergised with Xyn10D and XT6 on WAX. In contrast, Xyn10D and FAE-1 DS values were gradually decreasing from 0.8 to 0.6, which suggest that both enzymes did not display synergy during the hydrolysis of OX (Fig. 4c). The reducing sugars also decrease by about twofolds from 2.95 mg (100% Xyn10D) to 1.7 mg (25%Xyn10D:75%FAE-1).

#### GH 11 (Xyn2A) and FAE-1 synergy

**Fig. 5:**
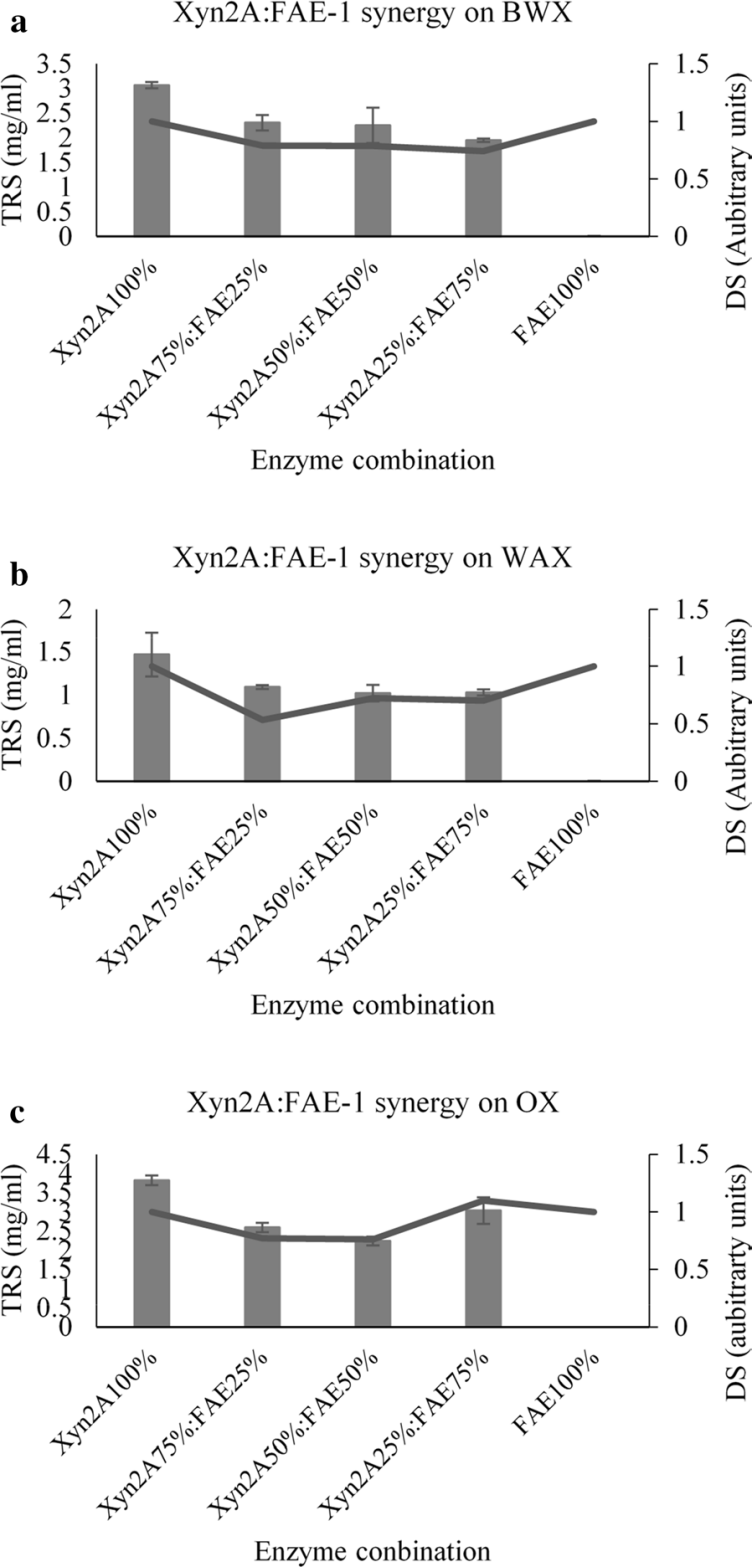
Synergistic hydrolytic activities of the GH11 xylanase (Xyn10D) and feruloyl esterase (FAE-1) on beechwood xylan (**a**), insoluble wheat arabinoxylan (**b**) and oat spelt xylan (**c**). The TRS represent the amount of released total reducing sugars from xylan and DS represents the degree of synergy formed between two enzymes. ANOVA analysis indicated the improvement of reducing sugar release by the enzyme combinations compared to lone enzyme protein loading, keys: *(p value < 0.05) and #(p value < 0.01). Values are represented as mean values ± SD (n = 3)

All the combinations of Xyn2A and FAE-1 resulted in no positive DS when the enzymes hydrolysed BWX and WAX (Fig. 5a, b). In addition, the combined hydrolytic activities of Xyn2A and FAE-1 released lower amounts of reducing sugars compared to those released by 100% Xyn2A enzyme load during the hydrolysis of BWX and WAX. These findings demonstrated that the two enzymes (Xyn2A and FAE-1) did not work synergistically on these substrates. The enzyme load of 25% Xyn2A to 75% FAE-1 displayed a DS value above 1 during the hydrolysis of OX, which suggested that these enzymes displayed synergy (Fig. 5c). However, the released reducing sugars were still lower (3.03 mg/ml) compared to those released by 100% Xyn2A (3.8 mg/ml). These findings reveal that the synergy between Xyn2A and FAE-1 did not result in an improved substrate degradation.

## Discussion

FAE enzyme activity was detected by the release of *p*-nitrophenol from *p*NP-substrates such as *p-*NP-ferulate by Mastihuba et al. (2002). The authors demonstrated that the enzymatic release of *p*-nitrophenol can be measured spectrophotometrically in the case where FAE activity is tested. Faiz et al. (2007) used pNP-butyrate to demonstrate that the *Anoxybacillus gonensis* A4 secreted an enzyme with esterase activity. The FAE-1 derived from the termite hindgut did not show activity on the tested xylan substrates, but it displayed high activity on *p*-NP-acetate. Mkabayi et al. (2020) demonstrated that FAEs from family 5 and 6 (referred to as FAE-5 and FAE-6) had negligible or no activity on WAX and corncob. The activity of FAE-5 and FAE-6 on WAX and CC were measured by reducing sugars released during substrate hydrolysis. These findings were interesting because FAE-1, FAE-5 and FAE-6 were sourced from the same termite (*Trinervitermes trinervoides*) hindgut bacteria. In addition, the *A. gonensis* A4 secreted FAE that displayed a pH optimum between pH 5.5 and 6, and temperature optima at 60 and 80 ℃ (Faiz et al. 2007). A recombinant FAE from *Talaromyces cellulolyticus* hydrolysed *p*NP-acetate and *p*NP-ferulate, and demonstrated pH optimum of 4.5–6 and temperature optimum of 65 ℃. The current study shows that the FAE-1 derived from a bacteria in the termite hindgut displayed temperature optimum that was consistent with that of FAEs sourced from fungi, except that it displayed relatively higher activity in the neutral pH range. It is important to note that some FAEs from other fungi such as *Aspergillus niger* BE-2 showed optimum temperature of about 45 ℃, which suggest that FAE-1 could be more stable at higher temperatures (50–70 ℃) than the *A. niger* BE-2 FAE (Wu et al. 2017).

The current study’s findings were consistent with literature with regards to Xyn2A because it’s more active on the less substituted xylan such as BWX and OX (Malgas et al. 2019; Faulds et al. 2003). However, the Xyn10D and XT6 results were unexpected since these enzymes displayed low activity on the insoluble-WAX, which was highly substituted (Fig. 2a). According to Collins et al. (2005) and Faulds et al. (2003), GH10 xylanase can only cleave at the third glycosidic bonds after a substituted residue, which suggests that Xy10D and XT6 activity on the WAX was affected by the arabinose substitution (mono or disubstitution) of the xylan backbone [3 arabinose substitutions per 5 xylose backbone units (Kiszonas et al. 2013 and Megazyme Lot 120801c; CAS No. 9040-27-1)]. However, Xyn10D and XT6 were highly active on BWX and OX compared to WAX.

Several studies have investigated the FAE enzymes or FAE and xylanase synergy capacity for the release of ferulic acid, *p*-coumaric acid and xylooligosaccharides from cereal crop residues such as maize, wheat and oat (MKabayi et al. 2020; Yu et al. 2002; Wu et al. 2017). However, the synergy between FAE and xylanase for the deconstruction of NSPs such as xylan from various agricultural residues is still not well explored. Here we used measurement of the  degree of synergy, which is a powerful tool used to demonstrate the synergism between two or more enzymes, to understand FAE-1 and xylanase synergy (Mafa et al. 2020; Van Dyk and Pletschke 2012). Binary synergy assays were employed to establish whether xylanases (Xyn10D, XT6 and Xyn2A) and FAE-1 acted synergistically during the hydrolysis of BWX, WAX and OX. The results demonstrated that the xylanases from GH10 displayed synergy mainly on WAX hydrolysis, but no synergy was displayed during OX hydrolysis.

Long et al. (2020) demonstrated that the *Eupenicillium parvum* recombinant *EP*FAE-1 from carbohydrate esterase (CE) family 1 was able to release ferulic acid from wheat bran treated with dilute phosphoric acid. In addition, the *EP*FAE-1 and a xylanase from GH10 (referred to as *EP*XYN1) released about 60% of ferulic acid from the wheat bran. The purified *pg*FAE from *Panus giganteus* and a GH10 xylanase synergized and resulted in a higher release of ferulic acid of about 112.2 µg/mg substrate compared to 17.6 µg/mg substrate released by *pg*FAE alone from wheat bran (Wang et al. 2014). However, *pg*FAE and xylanase synergic activity released only 13.7 µg/mg ferulic acid from corn bran, which Wang et al. (2014) argued was due to differences in biomass chemistry. Similarly, the biomass chemistry and the substitution of the backbone of xylans affected the performance of FAE-1 and GH10 xylanases (Xyn10D and XT6). Synergism between FAE-1 and GH10 xylanases on BWX was not expected because BWX has glucuronic acid and acetyl substitutions. We are tempted to speculate that FAE-1 possess both acetyl and feruloyl esterase activities because it exhibited partial synergy with both Xyn10D and XT6 during the hydrolysis of BWX. The current study measured the synergy between the FAE-1 and GH10 (Xyn10D and XT6) xylanases by monitoring the amounts of total reducing sugars released from the various xylan substrates (Figs. 3 and 4). It appears that most of the studies if not all the studies that were done on the FAE and GH10 xylanase synergy only focused on the release of ferulic acid, which suggests that the current study could be the first to report the effect of FAE on the GH10 xylanases during the deconstruction of NSP.

The GH11 xylanase (Xyn2A) and FAE-1 did not display effective synergy during the hydrolysis of BWX, WAX and OX samples. XynA derived from *Thermomyces lanuginosus* and two FAE enzymes (FAE5 and FAE6) displayed synergy on WAX, untreated corn cobs, hydrothermal pretreated and acid pretreated corn cobs, resulting in approximately two-fold higher reducing sugars (Mkabayi et al. 2020). Oliveira et al. (2019) argued that several FAEs and debranching enzymes respond differently towards the feruloylated polysaccharides. It seems that this phenomenon also applies between the various types FAEs and xylanases because the FAEs from the same termite hindgut metagenome reacted differently (e.g. FAE-1 and GH10 xylanases synergised releasing higher amounts of reducing sugars from WAX, however, no synergy was displayed on OX).

The FAE-1 does not possess activity on sugars linked by glycosidic bonds, but it displayed a high esterase activity on the model substrate *p*-NP-acetate, suggesting that FAE-1 could hydrolyse the ester bonds between ferulic acids and arabinoxylan. In addition, we demonstrated that xylanases were highly active on the xylan substrate with lesser substitutions like BWX and OX, but their activity dropped significantly during the hydrolysis of a highly substituted xylan substrate, WAX. The synergy of the GH 10 xylanases (XT6 and Xyn10D) and FAE-1 resulted in higher degradation of WAX, no improvement on BWX and OX degradation, but FAE-1did does not exhibit synergy with GH11. These findings contributed to our understanding toward the use of FEA-1 in synergy with GH10 xylanases for hydrolysis of NSPs in the animal feed and baking industries.

## Data Availability

All the data is presented in the manuscript and any additional data will be provided upon request.
